# Bioconversion of Corticosterone into Corticosterone-Glucoside by Glucosyltransferase

**DOI:** 10.3390/molecules23071783

**Published:** 2018-07-19

**Authors:** Tokutaro Yamaguchi, Joo-Ho Lee, A-Rang Lim, Joon-Soo Sim, Eun-Ji Yu, Tae-Jin Oh

**Affiliations:** 1 Department of Pharmaceutical Engineering and Biotechnology, Sun Moon University, 70 Sunmoon-ro 221, Tangjeong-myeon, Asan-si, Chungnam 31460, Korea; yamaguchi@sunmoon.ac.kr; 2 Genome-based BioIT Convergence Institute, 70 Sunmoon-ro 221, Tangjeong-myeon, Asan-si, Chungnam 31460, Korea; shadowjhl@empal.com; 3 Department of Life Science and Biochemical Engineering, Sun Moon University, 70 Sunmoon-ro 221, Tangjeong-myeon, Asan-si, Chungnam 31460, Korea; yuego@naver.com; 4 Korea Institute of Oriental Medicine, 1672 Yuseongdae-ro, Yuseong-gu, Daejeon 305-811, Korea; lar747@kiom.re.kr; 5 Genomics Division, National Institute of Agricultural Science, RDA, Jeonju 54874, Korea; jssim@korea.kr

**Keywords:** corticosterone, enzymatic glucosylation, glucocorticoid, NMR, steroid

## Abstract

Glucosylation of the 21-hydroxyl group of glucocorticoid changes its solubility into hydrophilicity from hydrophobicity and, as with glucocorticoid glucuronides as a moving object in vivo, it is conceivable that it exhibits the same behavior. Therefore, glucosylation to the 21-hydroxyl group while maintaining the 11*β*-hydroxyl group is particularly important, and glucosylation of corticosterone was confirmed by high-resolution mass spectrometry and 1D (^1^H and ^13^C) and 2D (COSY, ROESY, HSQC-DEPT and HMBC) NMR. Moreover, the difference in bioactivity between corticosterone and corticosterone 21-glucoside was investigated in vitro. Corticosterone 21-glucoside showed greater neuroprotective effects against H_2_O_2_-induced cell death and reactive oxygen species (ROS) compared with corticosterone. These results for the first time demonstrate that bioconversion of corticosterone through the region-selective glucosylation of a novel compound can present structural potential for developing new neuroprotective agents.

## 1. Introduction

Drugs, poison, or in vivo waste materials are generally metabolized by cytochrome P450 in the liver and excreted in the urine. Some hydrophobic compounds turn into hydrophilic compounds through a conjugation reaction with glutathione or glucuronic acid using UDP-glucuronosyl-transferase (UGT), and finally these metabolites are excreted through the bile duct or blood [1,2,3]. The steroidal hormones are secreted mainly in the testes, ovary, adrenal gland, gonad, and erasion endocrine glands by an internal factor and external stimulation, and are then synthesized in the adrenal glands and carried to the target organs [4,5]. In general, the steroidal hormones are classified into androgen, estrogen and corticoid by physiological function and structure [6,7,8,9,10,11,12,13]. Particularly, the corticoid hormones are further classified into glucocorticoid and mineralocorticoid. Furthermore, examples of glucocorticoids include corticosterone, cortisone and cortisol synthesized in the adrenal gland. Corticosterone is a main adrenal cortical hormone and is secreted only in human beings in a small quantity with a low level of activity.

Because glucocorticoids express physiological functions such as immune [14,15,16,17,18,19], growth [20,21,22], emotions [23,24,25] and recognition [26,27,28], they can be used as a drug with anti-inflammatory and immunity control action effects [14]. Research and development of steroidal hormones or innovative drugs having various in vivo effects are important, and the synthesis of the standard reagents and trial manufacture medicines are essential to studies of the properties and actions of these hormones. In particular, oxidative stress, bioenergetics impairment, and mitochondrial failure have been implicated in neurodegenerative diseases such as Alzheimer’s disease, Parkinson’s disease, and stroke [29]. A stress-induced increase in corticosterone secretion is known to produce neuronal cell damage, and may correlate with behavioral manifestations of depression [30]. Corticosterone is released into the bloodstream during experiences of stress, readily enters the brain through the blood-brain-barrier, and is distributed throughout different brain areas [31].

A majority of glucuronides have glucuronic acid linked to a hydroxyl group of the 21st carbon [32]. Corticoid glucuronides are useful in vivo, and the data of the synthetic methods, the melting point, the optical rotation properties, infrared spectrometers (IR), ultraviolet-visible spectrometry (UV), and the Nuclear Magnetic Resonance (NMR) are known [29,30,31,32,33,34,35,36]. However, cortisol and corticosterone which are adrenal cortical hormones are lipids, and thus they have to undergo an anhydrous reaction or a number of steps, and the purchase of cortisol and corticosterone is difficult. As with steryl, sterol, and steroid glucosides being commonly discovered from plants and bacteria, corticoid glucosides are advantageous in terms of having a higher hydrophilicity [37,38,39,40,41], and there have been a number of reports about the use of corticoid glucosides in vivo research for drug development [42,43]. Glycosylation occurring at oxygen binding to the 11*β*-hydroxyl group of corticoid has been reported [44], but there have been no reports about glycosylation occurring at oxygen binding to the 21-hydroxyl group. Meanwhile, 11-deoxycorticosterone moieties only have a limited bondable position of the 21-hydroxyl group, and compounds obtained by performing acetylation and glucoside compounds at the 21-hydroxyl group of 11-deoxycorticosterone moieties have been used for many in vivo studies [45,46,47,48]. However, such compounds have a limitation on the glycosylation position, and oxygen binds only to the 21-hydroxyl group. Therefore, compounds obtained by performing glycosylation only at the 21-hydroxyl group while leaving the 11*β*-hydroxyl group alone are considered to be very useful for the aforementioned reasons. In addition, the NMR structure elucidation of corticosterone and its derivatives is poor in comparison with cortisone and cortisol.

In this study, glucosyltransferase was used to perform glucosylation at the 21-hydroxyl group of corticosterone while leaving the 11*β*-hydroxyl group alone, and corticosterone glucoside structure was elucidated by one-dimensional (1D) NMR of ^1^H and ^13^C, 2D of COrrelated SpectroscopY (COSY), Rotating-frame Overhauser Effect SpectroscopY (ROESY), Heteronuclear Single-Quantum Correlation-Distortionless Enhancement by Polarization Transfer (HSQC-DEPT), Heteronuclear Multiple-Bond Correlation (HMBC) NMR experimentation, and mass spectrometry. After the large-scale preparation and isolation of corticosterone 21-glucoside, we investigated its neuroprotective activity on H_2_O_2_-induced SH-SY5Y neuroblastoma cells and compared with that of corticosterone.

## 2. Results and Discussion

### 2.1. Bioconversion of Corticosterone 21-Glucoside

**Figure 1:**
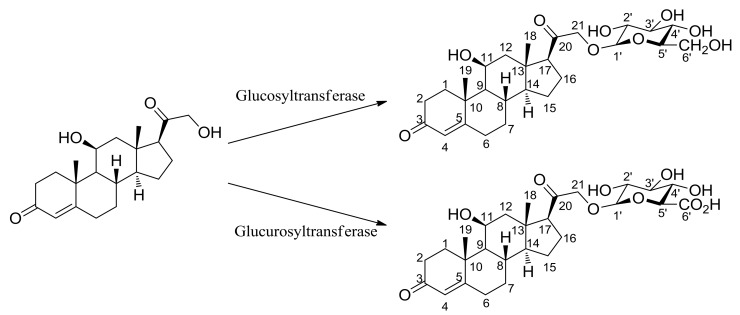
The glucosyltransferase and glucurosyltransferase [32] reaction scheme of corticosterone.

**Figure 2:**
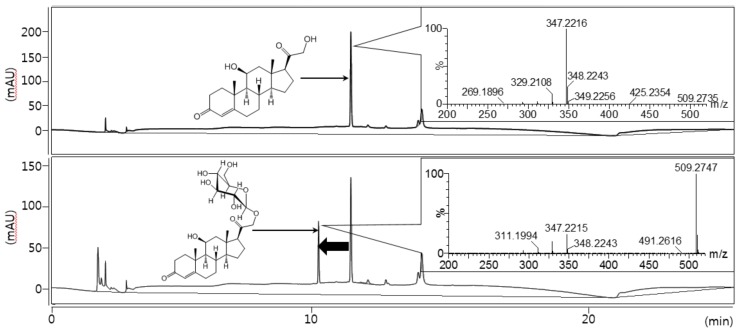
HPLC analysis of the glucosylated corticosterone product after biotransformation.

As a result of performing biotransformation (Figure 1) for corticosterone in which about 40% conversion yields were obtained, an HPLC analysis of the corticosterone reaction mixture showed only one glucoside peak at 254 nm. The time of flight electro-spray high resolution mass spectrometry (TOF-ESI HRMS) of this product showed *m*/*z* = 509.2747 (C_27_H_41_O_9_^+^: a calculated exact mass value *m*/*z* = 509.2745) which is consistent with the [M + 1]^+^ exact mass of corticosterone glucoside (Figure 2).

NMR experimentation measured ^1^H, ^13^C (Figure S1), COSY, ROESY, HSQC-DEPT, and HMQC (Figure S2) and identified carbon with each hydrogens (Table S1) and the steric structure by ROESY. The ^1^H- and ^13^C-NMR chemical shifts of corticosterone 21-glucuronide were reported by Ciuffreda et al. [35]. Corticosterone 21-glucuronide and corticosterone 21-glucoside are almost identical in terms of the aglycon portion, and the only difference is carboxylic acid and alcohol in the 6′ positions of the sugar moiety as shown in Figure 1. A comparison corticosterone derivative between glucuronide and glucoside for chemical shifts of ^13^C and ^1^H and ^1^H-^1^H coupling constants is shown in Table 1.

It followed that the ^13^C and ^1^H chemical shift of aglycone moiety almost accords with the results of Ciuffreda et al*.*, however the chemical shift of H-12 as *α* and *β* demonstrated the reverse of these results [35]. The decision of *α* and *β* in the steroid was determined to act as follows: When the rings of a steroid are denoted as projections on the plane of the paper, the formula is normally oriented as in Figure 1 [49]. An atom or group attached to a ring depicted in the orientation in Figure 1 is termed *α* (alpha) if it lies below the plane of the paper or *β* (beta) if it lies above the plane of the paper [34]. These *α* and *β* positions can be decided by investigating the steric correlation, using the method of observing the Nuclear Overhauser Effect (NOE) between each hydrogens. What ROESY or NOESY experiment is desirable due to being complexed by overlapping the ^1^H-NMR signal of other aglycone protons.

**Figure 3:**
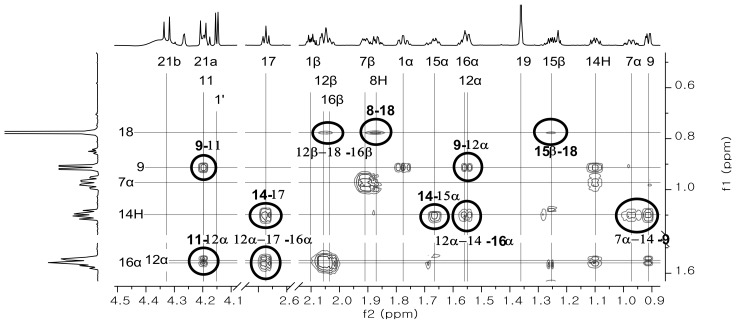
The partial ROESY spectra of corticosterone 21-glucoside for assignment of aglycone moiety H-12 and other hydrogens bound to a sterocenter. The cross peak in the circle concerned is a notable peak, and the numerical value at the side shows a position number of hydrogen at aglycone.

**Figure 4:**
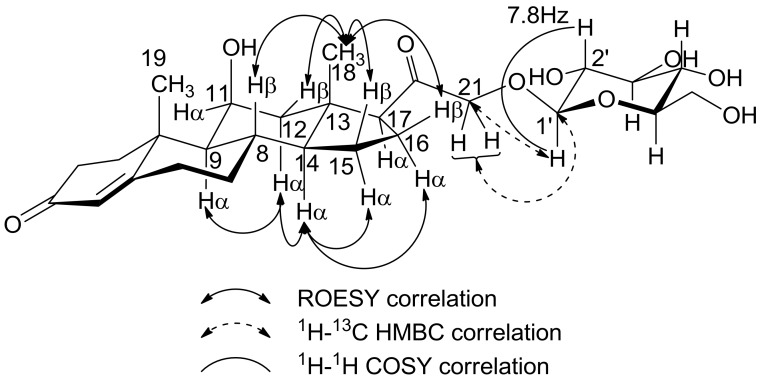
Corticosterone 21-glucoside shape and ^1^H-^1^H correlation of NOE for stereochemistry confirmations at H-9, H-14 and H-18 are determined ROESY. In addition, the glucose linkage position with corticosterone was indicated by the investigation of ^1^H-^13^C long-range coupling correlation using ^1^H-^13^C HMBC experiment. The determination of the glucose moiety anomericity was presented by the elucidation of ^1^H-^1^H COSY and ^1^H-^13^C HMBC experiments.

The result of ROESY experimentation indicated that the correlated NOE cross peaks of each of the steric positioning hydrogens (Figure 3) H-9 and H-14 at *δ* 0.91 and *δ* 1.10, respectively, are in the axial and *α* position, therefore *δ* 1.55 of H-12, *δ* 1.67 of H-15, *δ* 1.56 of H-16 and *δ* 0.97 of H-7 should be at the *α* position because of the indicated NOE cross peaks. In the case of NOE experiment results, there is a higher possibility for the NOE correlation to be between H-12, H-15, H-16 and H-8 at *δ* = 2.05, 1.25, 2.03 and 1.87, respectively, in H-18 at *δ* 0.78. This possibility was indicated practically in Figure 3. These NOE correlations were shown as the structural scheme in Figure 4.

The aglycone of corticosterone glucoside identified each proton and carbon. Because of the two hydroxyl groups in corticosterone, the linkage position must be determined among 11*β*- and the 21-hydroxyl group. If the glucose is linked to the 11*β*-hydroxyl group, the chemical shifts of H-11 and H-21 should show different values for glucuronide and glucoside in Table 1. When the chemical shifts of glucuronide and glucose were compared in Table 1, the results for glucoside show the same connected position of glucose to corticosterone. Moreover, the position of the glycosidic linkage of the steroid glucoside was confirmed by the inter-glycosidic correlation in the ^1^H-^13^C HMBC spectra in which the correlation between signals at *δ* 4.15 (H-1′) and *δ* 73.44 (C-21) confirmed the substitution at the C-21 position of the aglycone by a *β*-glucoside moiety. Also, correlation of HMBC spectra between signals at *δ* 4.20 and 4.33 (H-21 two hydrogen) and *δ* 102.14 (C-1′) confirmed the above linkage position.

Confirmation of the anomericity of the glucose moiety in corticosterone 21-glucoside was obtained by 1D ^1^H-NMR after assignment of the anomeric proton and H-2′ by 2D COSY experimentation. The relatively large coupling constant between the H-1′ and H-2′ values of the glucose moiety (7.8 Hz, Figure 4 and Table 1) indicated the *β*-conformation on the D-glucose moiety [35] (Table 1).

### 2.2. Bioactivity of Corticosterone 21-Glucoside

**Figure 5:**
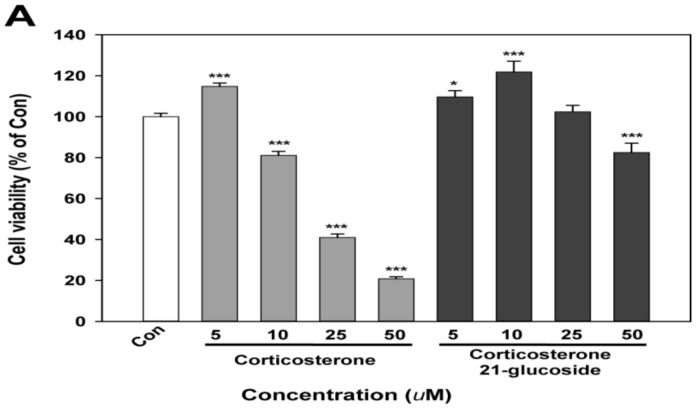

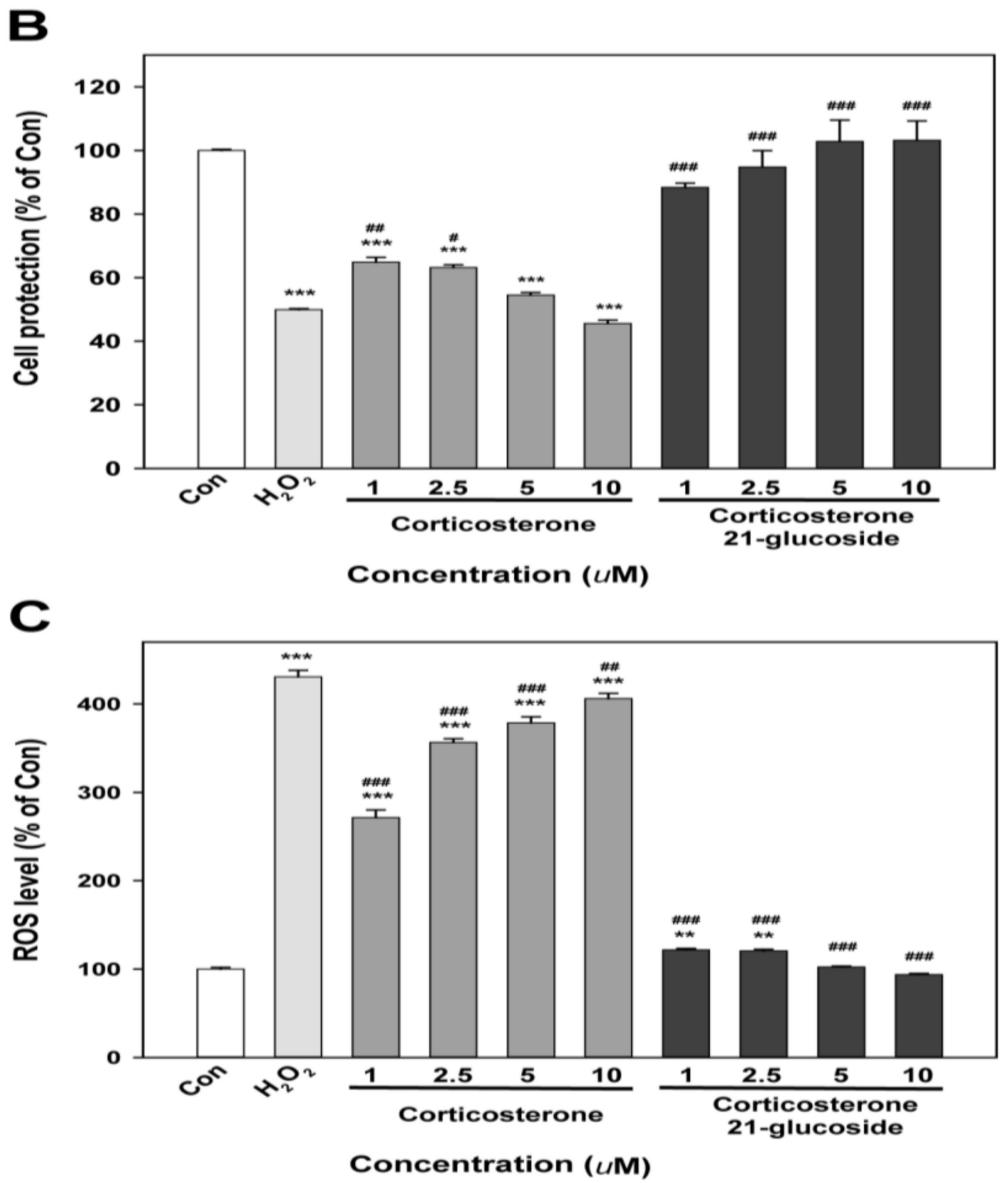
Effects of corticosterone and corticosterone 21-glucoside on SH-SY5Y neuroblastoma cell line. (**A**) Cell viability; (**B**) Cell protection on H_2_O_2_ treated cells; (**C**) Reactive oxygen species (ROS) production on H_2_O_2_ treated cells. Data are reported as percentages of the control. * *p* < 0.05, ** *p* < 0.01 and *** *p* < 0.001 compared with the result of control. ^#^ *p* < 0.05, ^##^ *p* < 0.01 and ^###^ *p* < 0.001 compared with that of H_2_O_2_ treated cells.

Neurodegenerative disorders are closely related to abnormal neuronal cell death [50], and neuronal cells in the brain are highly sensitive to oxidative stress due to their larger dependence on oxidative phosphorylation for energy compared to other cells [51]. As a stress hormone, corticosterone leads to neuronal injuries characterized by DNA damage and cell apoptosis. In this study, we investigated the neuroprotective activity of corticosterone and corticosterone 21-glucoside against H_2_O_2_-induced cell death and reactive oxygen species (ROS) on SK-N-SH cells. In addition, we first investigated the difference in cytotoxicity between corticosterone and corticosterone 21-glucoside. As shown in Figure 5A, corticosterone treatment (5, 7.5, 10, 25 and 50 µM) dramatically decreased cell viability by 114.8%, 98.8%, 81.1%, 41.0% and 20.8%, respectively, compared with the control (100%). However, cytotoxicity by corticosterone 21-glucoside (5, 7.5, 10, 25 and 50 µM) was 109.6%, 121.5%, 121.9%, 102.4% and 82.5%, respectively, showing that it was not severe compared with that of corticosterone (Figure 5A).

In order to test the neuroprotective activity of corticosterone and corticosterone 21-glucoside, a cell protection assay was carried out on the cells with/without 100 µM H_2_O_2_. When we treated 100 µM of H_2_O_2_ to SK-N-SH cells, significant cytotoxicity (49.9% survival rate compared with the control) was shown. Pretreatment with corticosterone 21-glucoside (1, 2.5, 5 and 10 µM), rescued cell viability by 88.4%, 94.8%, 102.9% and 103.2%, respectively, compared with (64.9%, 63.2%, 54.5% and 45.6%, respectively) corticosterone (1, 2.5, 5 and 10 µM) (Figure 5B). Corticosterone 21-glucoside was superior to corticosterone in terms of inhibition of H_2_O_2_-mediated cytotoxicity in a dose-dependent manner (Figure 5B).

Neurodegenerative diseases which are related to the disorder of mitochondrial metabolism conduct increased ROS generation and mitochondrial dysfunction [52]. The generation of mitochondrial superoxide is considered to play an important role in the degradation of cellular functioning [47]. Thus, we performed an ROS production assay to examine the mitochondrial superoxide scavenging activity of corticosterone and corticosterone 21-glucoside. Treatment of H_2_O_2_ to SK-N-SH cells increased the production of ROS to 430.7% compared with that of the control (100%) (Figure 5C). Pretreatment with corticosterone 21-glucoside (1, 2.5, 5 and 10 µM) significantly reduced mitochondrial superoxide levels by 121.6%, 120.6%, 102.4% and 93.8%, respectively, compared with (271.5%, 356.6%, 378.6% and 406.1%, respectively) corticosterone (1, 2.5, 5 and 10 µM) (Figure 5C). Similar to the results of the neuroprotective activity test, corticosterone 21-glucoside exhibited a much greater activity in the down-regulation of mitochondrial superoxide levels than corticosterone in a dose-dependent manner (Figure 5C).

## 3. Materials and Methods

### 3.1. General Experimental Procedures for Corticosterone 21-Glucoside

#### 3.1.1. Isolation of Corticosterone 21-Glucoside

The product was analyzed by a Dionex Ultimate 3000 UHPLC+ system (Thermo Fisher Scientific, Germering, Germany) using a Mightysil reverse-phase C18 GP column (4.6 × 250 mm, 5 μm). The configuration of the HPLC system consisted of an LPG-3400SD pump, ACC-3000 auto-sampler column compartment, and DAD-3000 diode array detector. The mobile phase consisted of solution A (in HPLC-grade water) and solution B (in HPLC-grade acetonitrile). The flow rate was maintained at 1.0 mL/min, and the oven temperature was kept at 30 °C. To analyze the products, the gradient system was operated under the following conditions: percentages of solution B were increased from 5% to 8% (0–4 min), 20% (7 min), 40% (10 min), 70% (13 min), 100% (18–25 min), 80% (28 min), 50% (30–33 min) and 5% (38 min). UV detection was performed at 245 nm to confirm the substrates and their products.

HPLC isolation: The purification of compounds was carried out by preparative (prep)-HPLC with a C18 column (YMC-Pack ODS-AQ (250 mm × 20 mm I.D., 10 μm)) connected to a UV detector (245 nm) using a 35 min binary program with ACN 5% to 40% (0–10 min), then, increased to 100% (10–18 min), kept at 100% (18–25 min), and decreased to 5% (25–35 min) at a flow rate of 10 mL/min.

#### 3.1.2. Nuclear Magnetic Resonance and Mass Spectrometry

The sample was prepared by dissolving the purified product in hexadeuterio-dimethyl-sulfoxide (DMSO-*d*_6_) on a Bruker BioSpin AVANCE II 900 MHz spectrometer (Bruker GmbH, Rheinstetten, Germany). One-dimensional (^1^H NMR and ^13^C NMR) and two-dimensional NMR (COSY, ROESY, HSQC-DEPT, and HMBC) tests were performed to elucidate the exact structure of the glucosylated steroid.

The masses of the in vitro glucosylation reaction starting substrate and product were confirmed by ultra-high performance liquid chromatography electro-spray ionization quadrupole time of flight high resolution mass spectrometry (UPLC-ESI-Q-TOF-HRMS) analysis using ACQUITY UPLC^®^ (Waters Corporation, Milford, MA, USA) coupled with SYNAPT G2-S (Waters Corporation).

### 3.2. Bioconversion Reaction

We found a UDP-glucosyltransferase gene (UGT-1) in the *Terribacillus* sp. PAMC 23288 obtained from an Arctic soil sample (unpublished data). UGT-1 gene is cloned into pET28a(+) and over-expressed in *Escherichia coli* BL21(DE3). Purification of UGT-1 was performed as generally protein purification method. For the in vitro assay, a final volume of 100 μL was constituted with 10 ug/mL glucosyltransferase, 100 mM Tris-HCl, pH 8.5 buffer, 10 mM MgCl_2_, 2 mM UDP-glucose and 0.4 mM corticosterone. The reaction proceeded for 3 h at 35 °C. When the reaction was terminated, the sample was extracted with 400 μL of methanol and centrifuged at 12,000× *g* for 10 min. The separated supernatant was used for further analysis.

The preparative-scale reaction was carried out in a 30 mL volume with purified glucosyltransferase (30 μg/mL), 10 mM UDP-glucose (~56 mg), 10 mM substrate (~10 mg, dissolved in DMSO), 100 mM Tris-HCl (pH 8.0) buffer and 10 mM MgCl_2_·6H_2_O, and it was incubated for 3 h at 37 °C. The reaction was stopped by adding a triple volume of chilled methanol. The reaction mixture briefly mixed and was centrifuged (12,000 rpm, 10 min, 4 °C) to remove denatured proteins. Finally, the supernatant was concentrated by evaporation and purification.

### 3.3. Cell Assay for Corticosterone 21-Glucoside

#### 3.3.1. Cell Cytotoxicity and Protection Assay

The SH-SY5Y neuroblastoma cell line was purchased from the American Type Culture Collection (Manassas, VA, USA). Cells were cultured in Dulbecco’s Modified Eagle’s Medium (Gibco-BRL, Karlsruhe, Germany) containing 10% fetal bovine serum (Gibco-BRL) supplemented with penicillin (100 U/mL) and streptomycin (100 µg/mL) at 37 °C in a humidified 5% CO_2_ incubator.

In order to test cell viability by the treatment of corticosterone and corticosterone 21-glucoside, cells were seeded on 96-well plates at a density of 1 × 10^4^ cells/well and were incubated at 37 °C for 24 h. The plates were treated with different concentrations (0, 5, 10, 25 and 50 µM) of corticosterone and corticosterone 21-glucoside and then additional incubation was performed at 37 °C for 24 h. Cell viability was determined by CellTiter 96^®^ Aqueous One Solution Cell Proliferation Assay kit (Promega, Madison, WI, USA) based on the reduction of [3-(4,5-dimethylthiazol-2-yl)-5-(3-carboxymethoxyphenyl)-2-(4-sulfophenyl)-2*H*-tetrazolium inner salt (MTS)] to formazan according to manufacturer directions. After removing media, 200 μL of DMEM containing MTS was added to each well and then incubated at 37 °C for 1 h. Absorbance was measured at 490 nm using a microplate fluorometer (Molecular Devices, Sunnyvale, CA, USA).

For checking the cell protection effect of corticosterone and corticosterone 21-glucoside, cells were seeded on 96-well plates at a density of 1 × 10^4^ cells/well and maintained at 37 °C for 24 h. Different concentrations (0, 1, 2.5, 5 and 10 µM) of corticosterone and corticosterone 21-glucoside were added to cells and incubated at 37 °C for 24 h. One hundred micromoles of hydrogen peroxide (H_2_O_2_) was added to cells and after incubation for 1 h, the cell protection effect was determined via reduction of MTS to formazan as previously described.

#### 3.3.2. Reactive Oxygen Species (ROS) Production Assay

Intracellular ROS was measured using a 5(6)-carboxy-2′,7′-dichlorofluorescein diacetate (DCF-DA, Sigma-Aldrich, St. Louis, MO, USA) fluorescent probe. SH-SY5Y cells were seeded to a 96-well black plate and test samples with different concentrations (0, 1, 2.5, 5 and 10 µM) were treated respectively for 24 h. One hundred micromoles of H_2_O_2_ was added to cells and incubated for 1 h. Cells were incubated with 10 µM of DCF-DA at 37 °C for 30 min and then washed twice with PBS. Fluorescence intensity of DCF was measured in a microplate reader with an excitation of 485 nm and an emission of 535 nm.

## 4. Conclusions

The structure and position of the glucosylation of corticosterone 21-glucoside were confirmed by ^1^H, ^13^C, COSY, ROESY, HSQC-DEPT, and HMQC NMR experiments and TOF-ESI HRMS data. Aside from the 11*β*-hydroxyl groups, it clarified the effectiveness of glucosylation if 21-hydroxyl groups. This almost corresponded with prior NMR research results [35], except for the difference between glucuronic acid and glucose in comparison. However, the present identification for hydrogen *α*–*β* configuration on C-12 of aglycone moiety turned out to be the reverse. Decision of stereochemistry using NOE is important in terms of stereocenter. However, in the previous reports, the steric configuration has not been determined by NOE, which brings about this difference. Thus far, no detailed NMR data of corticosterone have been reported, but this NMR analysis is considered to supplement the structure data of the steroid.

Cytotoxicity of corticosterone 21-glucoside was not severe (109.6–82.5% vs. the control) when compared with corticosterone (114.8–20.8% vs. the control) at the different concentrations (5–50 µM). Pretreatment with corticosterone 21-glucoside (1–10 µM) rescued cell viability (88.4–103.2% vs. the control) and decreased intracellular ROS level (121.6–93.8% vs. the control, 430.7% in H_2_O_2_ treatment) compared with corticosterone (64.9–45.6% in cell viability and 271.5–406.1% in ROS level, respectively) in the presence of 100 µM H_2_O_2_. These results showed that corticosterone 21-glucoside attenuated cell toxicity in comparison to corticosterone. The observed neuroprotective effects of corticosterone 21-glucoside suggest that this compound may be useful for achieving selective neuroprotective action.

## Figures and Tables

**Table 1:** Comparison of ^13^C- and ^1^H-NMR chemical shifts (ppm) and coupling constants (Hz) of corticosterone-21-glucuronide and 21-glucoside.

^13^C			^1^H with Coupling Constants *J*_HH_^1^				
	21-Glucuronide ^2^	21-Glucoside ^3^		21-Glucuronide ^2^		21-Glucoside ^3^	
	*δ* (ppm)	*δ* (ppm)		*δ* (ppm)	*J*_HH_ (Hz)	*δ* (ppm)	*J*_HH_ (Hz)
Aglycone moiety			Aglycone moiety				
1	34.45	34.04	1*α*	1.77	13.2, 4.0	1.78	ddd, 13.6, 13.6, 4.4
			1*β*	2.10	3.4, 3.1	2.10	ddd, 13.3, 4.7, 4.7
2	33.93	33.48	2*α*	2.19	4.0, 3.4	2.18	ddd, 16.8, 4.0, 4.0
			2*β*	2.39	13.2, 3.1	2.38	ddd, 16.5, 13.8, 5.0
3	198.52	198.1					
4	121.97	121.52	4	5.63		5.56	d, 1.7
5	172.73	172.32					
6	31.79	31.35	6*α*	2.17	13.0, 4.3, 2.1	2.18	ddd, 16.8, 4.0, 4.0
			6*β*	2.44	13.2, 13.0. 5.0	2.44	dddd, 14.2, 14.2, 5.6, 1.6
7	32.95	32.52	7*α*	0.98	13.2, 12.6, 11.2, 4.3	0.97	dddd, 14.6, 12.6, 11.2, 4.6
			7*β*	1.87	12.6, 5.0, 3.5, 2.1	1.91	dddd, 12.2, 5.8, 4.1, 2.2
8	31.55	31.13	8	1.92	12.2, 11.2, 11.2, 3.5	1.87	dddd, 11.2, 11.2, 11.1, 4.1
9	55.95	55.46	9	0.91	12.2, 4.1	0.91	dd, 11.2, 3.4
10	39.44	38.86					
11	66.67	66.14	11*α*	4.21	4.1, 4.1, 2.7	4.20	dddd, 9.7, 3.3, 3.2, 3.2
			11*β*	-		-	
12	46.96	46.52	12*α*	2.02	12.7, 4.1	1.57–1.53	m
			12*β*	1.54	12.7, 2.7	2.07–2.04	m
13	43.82	43.41					
14	57.33	56.91	14	1.09	11.2, 6.3	1.10	ddd, 12.5, 10.6, 7.1
15	24.54	24.13	15*α*	1.66	12.4, 12.4, 6.3, 2.2	1.67	dddd, 12.1, 9.7, 7.1, 2.9
			15*β*	1.25	12.4, 11.2, 11.2, 5.8	1.25	dddd, 12.1, 12.1, 12.0, 6.6
16	22.29	21.8	16*α*	1.57	13.6, 12.4, 8.4, 5.8	1.59–1.53	m
			16*β*	2.04	13.6, 11.2, 8.4, 2.2	2.03	ddd, 13.5, 9.3, 2.9
17	58.51	58.06	17	2.69	8.4, 8.4	2.67	t, 9.2
18	16.17	15.76	18-CH_3_	0.79		0.78	s
19	20.82	20.37	19-CH_3_	1.37		1.36	s
20	207.79	207.61					
21	74.56	73.44	21	4.29	18.2	4.33	d, 17.3
			21	4.16	18.2	4.20	d, 17.3
Sugar moiety			Sugar moiety				
1′	103.2	102.14	1′	4.28	7.8	4.15	d, 7.8
2′	73.48	73.30	2′	3.07	8.6, 7.8	3.00	dd, 8.4, 8.4
3′	76.36	76.55	3′	3.18	8.6, 8.0	3.12	dd, 8.9, 8.9
4′	71.85	69.97	4′	3.33	9.5, 8.0	3.02	dd, 9.3, 9.3
5′	76.17	77.03	5′	3.63	9.5	3.08	ddd, 9.9, 6.1, 2.1
6′	170.71	61.09	6′			3.42	dd, 11.6, 6.1
			6′			3.66	dd, 11.7, 2.1

^1^ Assignments from ^1^H-^1^H COSY, ROESY, HSQC-DEPT and HMQC. An experimental error in the measured ^1^H-^1^H coupling constants was ±0.6 Hz. ^2^ Reference [35]. ^13^C NMR (125 MHz, DMSO-*d*_6_) and ^1^H NMR (500 MHz, DMSO-*d*_6_). ^3 13^C NMR (226 MHz, DMSO-*d*_6_) and ^1^H NMR (900 MHz, DMSO-*d*_6_).

## Supplementary Materials

The following are available online. Figure S1: ^1^H and ^13^C NMR analysis of corticosterone glucoside, Figure S2: 2D (COSY, ROESY, HSQC-DEPT and HMBC) NMR of corticosterone glucoside, Table S1: ^1^H-NMR and ^13^C-NMR chemical shifts^1^ (ppm) of corticosterone glucoside.
