# Supplementary material for: Bioconversion of Corticosterone into Corticosterone-Glucoside by Glucosyltransferase
Source: Molecules. 2018 Jul 19;23(7):1783. doi: 10.3390/molecules23071783 (PMC6100193; doi:10.3390/molecules23071783)

SUPPLEMENTARY MATERIAL

Molecules

Bioconversion of corticosterone into corticosterone-glucoside by glucosyltransferase

Tokutaro Yamaguchi [1,2,3,†], Joo-Ho Lee[2,†], A-Rang Lim[4], Joon-Soo Sim[5], Eun-Ji Yu[3], and Tae-Jin Oh[1,2,3,*]

[1] Department of Pharmaceutical Engineering and Biotechnology, Sun Moon University, 70 Sunmoon-ro 221, Tangjeong-myeon, Asan-si, Chungnam 31460, Republic of Korea; E-Mails: yamaguchi@sunmoon.ac.kr (T.Y.)
[2] Genome-based BioIT Convergence Institute, 70 Sunmoon-ro 221, Tangjeong-myeon, Asan-si, Chungnam 31460, Republic of Korea; E-Mail: shadowjhl@empal.com (J.H.L.)
[3] Department of Life Science and Biochemical Engineering, Sun Moon University, 70 Sunmoon-ro 221, Tangjeong-myeon, Asan-si, Chungnam 31460, Republic of Korea; E-Mail: yuego@naver.com (E.J.Y.); tjoh3782@sunmoon.ac.kr (T.J.O.)
[4] Korea Institute of Oriental Medicine, 1672 Yuseongdae-ro, Yuseong-gu, Daejeon, 305-811, Republic of Korea; E-Mail: lar747@kiom.re.kr (A.R.L.)
[5] Genomics Division, National Institute of Agricultural Science, RDA, Jeonju, 54874, Republic of Korea; E-Mail: jssim@korea.kr (J.S.S.)

† These authors contributed equally to this work.
* Author to whom correspondence should be addressed; E-Mail: tjoh3782@sunmoon.ac.kr ; Tel.: +82-41-530-2677; Fax:+82-41-530-2279.

Abstract: Glucosylation of the 21-hydroxyl group of glucocorticoid changes its solubility into hydrophilicity from hydrophobicity and, as with glucocorticoid glucuronides as a moving object in vivo, it is conceivable that it exhibits the same behavior. Therefore, glucosylation to the 21-hydroxyl group while maintaining the 11β-hydroxyl group is particularly important, and glucosylation of corticosterone was confirmed by high-resolution mass spectrometry and 1D (^{1}H and ^{13}C) and 2D (COSY, ROESY, HSQC-DEPT and HMBC) NMR. Moreover, the difference in bioactivity between corticosterone and corticosterone 21-glucoside was investigated in vitro. Corticosterone 21-glucoside showed greater neuroprotective effects against H_2O_2-induced cell death and reactive oxygen species (ROS) compared with corticosterone. These results for the first time demonstrate that bioconversion of corticosterone through the region-selective glucosylation of a novel compound can present structural potential for developing new neuroprotective agents.

Keywords: corticosterone; enzymatic glucosylation; glucocorticoid; NMR; steroid

Figure legends

Figure S1. ^{1}H and ^{13}C NMR analysis of corticosterone glucoside.

Figure S2. 2D (COSY, ROESY, HSQC-DEPT and HMBC) NMR of corticosterone glucoside.

Table S1. ^{1}H-NMR and ^{13}C-NMR chemical shifts[1] (ppm) of corticosterone glucoside

^{13}C		^{1}H with coupling constants J_{HH}[2]		
	δ(ppm)		δ(ppm)	J_{HH}(Hz)
Aglycon moiety				
1	34.04	1α	1.78	ddd, 13.6, 13.6.4.4
		1β	2.10	ddd, 13.3, 4.7, 4.7
2	33.48	2α	2.18	ddd, 16.8, 4.0, 4.0
		2β	2.38	ddd, 16.5, 13.8, 5.0
3	198.1			
4	121.52	4	5.56	d, 1.7
5	172.32			
6	31.35	6α	2.18	ddd, 16.8, 4.0, 4.0
		6β	2.44	dddd, 14.2, 14.2, 5.6, 1.6
7	32.52	7α	0.97	dddd, 14.6, 12.6, 11.2, 4.6
		7β	1.91	dddd, 12.2, 5.8, 4.1, 2.2
8	31.13	8	1.87	dddd, 11.2, 11.2, 11.1, 4.1
9	55.46	9	0.91	dd, 11.2, 3.4
10	38.86			
11	66.14	11α	4.20	dddd, 9.7, 3.3, 3.2, 3.2
		11β		
12	46.52	12α	1.57 – 1.53	m
		12β	2.07 – 2.04	m
13	43.41			
14	56.91	14	1.10	ddd, 12.3, 10.8, 7.1
15	24.13	15α	1.67	dddd, 12.1, 9.7, 7.1, 2.9
		15β	1.25	dddd, 12.1, 12.1, 12.0, 6.6
16	21.8	16α	1.59 – 1.53	m
		16β	2.03	ddd, 13.5, 9.3, 2.9
17	58.06	17	2.67	t, 9.2
18	15.76	18-CH_3	0.78	s
19	20.37	19-CH_3	1.36	s
20	207.61			
21	73.44	21	4.33	d, 17.3
		21	4.20	d, 17.3
Sugar moiety				
1'	102.14	1'	4.15	d, 7.8
2'	73.30	2'	3.00	dd, 8.4, 8.4
3'	76.55	3'	3.12	dd, 8.9, 8.9
4'	69.97	4'	3.02	dd, 9.3, 9.3
5'	77.03	5'	3.08	ddd, 9.6, 6.2, 2.1
6'	61.09	6'a	3.42	dd, 11.6, 6.1
		6'b	3.66	dd, 11.7, 2.1

[1] ^{13}C NMR (226 MHz, DMSO-*d6*) and ^{1}H NMR (900 MHz, DMSO-*d6*). Assingments from ^{1}H-^{1}H COSY, ROESY, HSQC-DEPT and HMQC. [2] Experimental error in the measured ^{1}H–^{1}H coupling constants was ± 0.6 Hz.

Figure S1.

^{1}H NMR

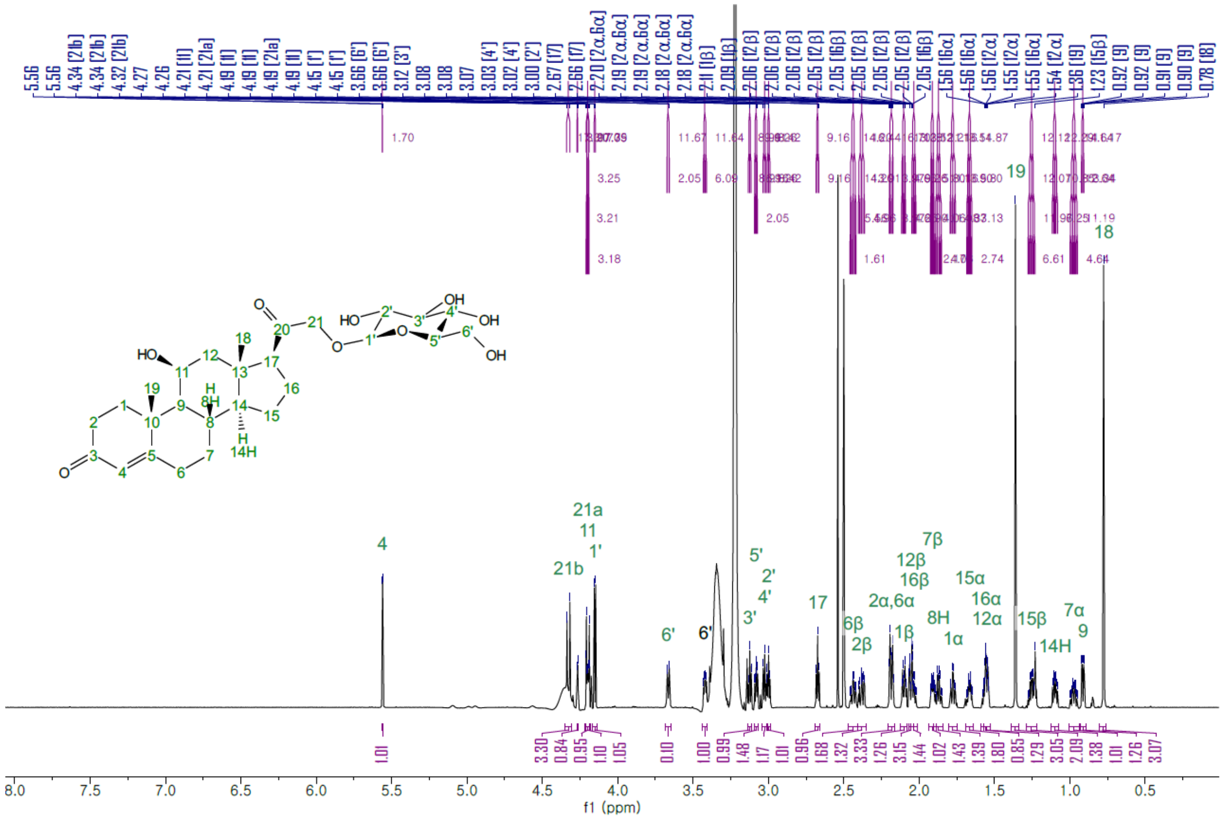

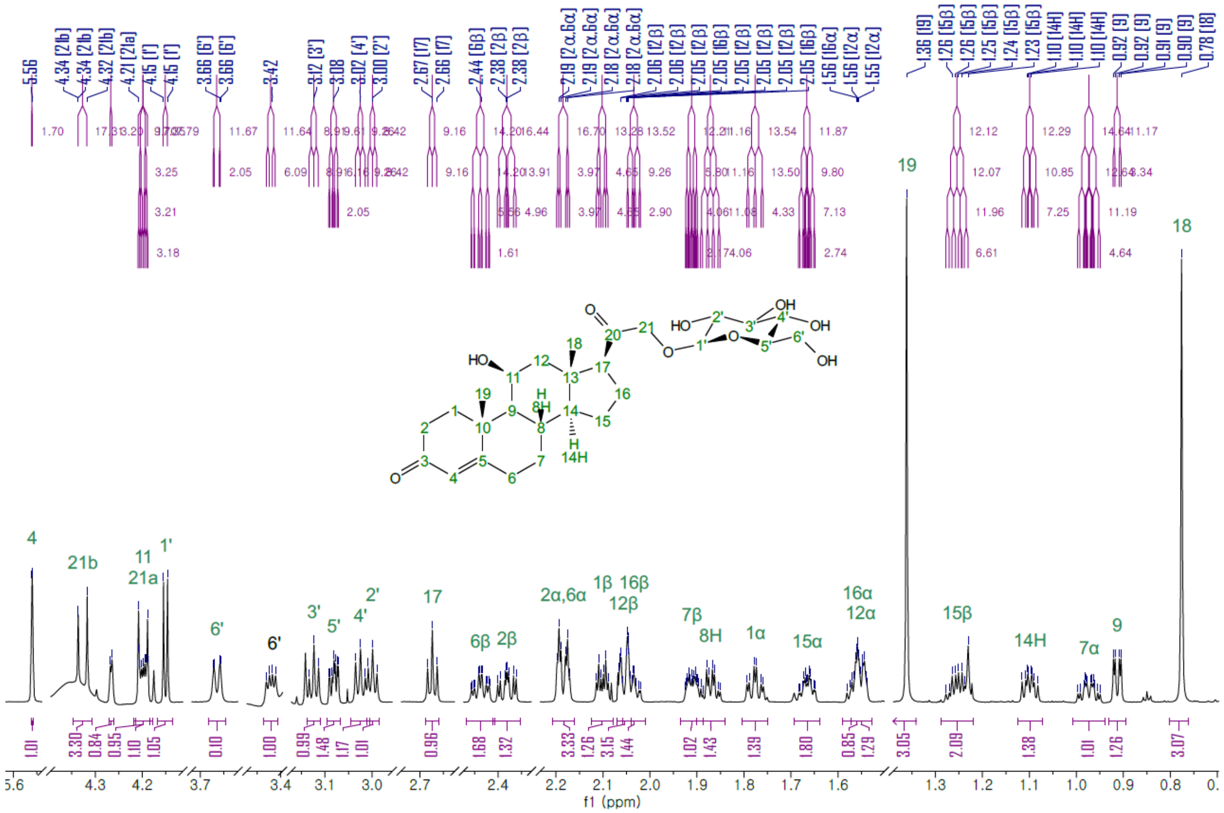

Figure S1.

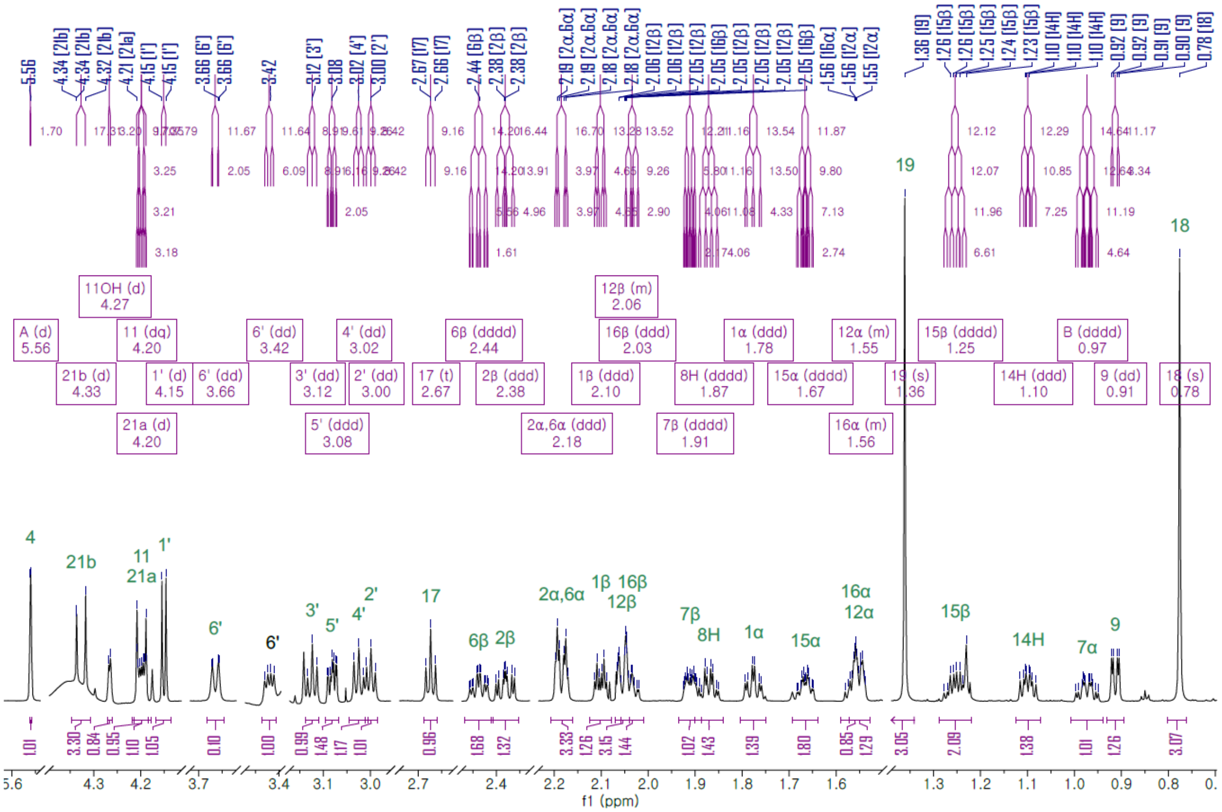

^{13}C NMR

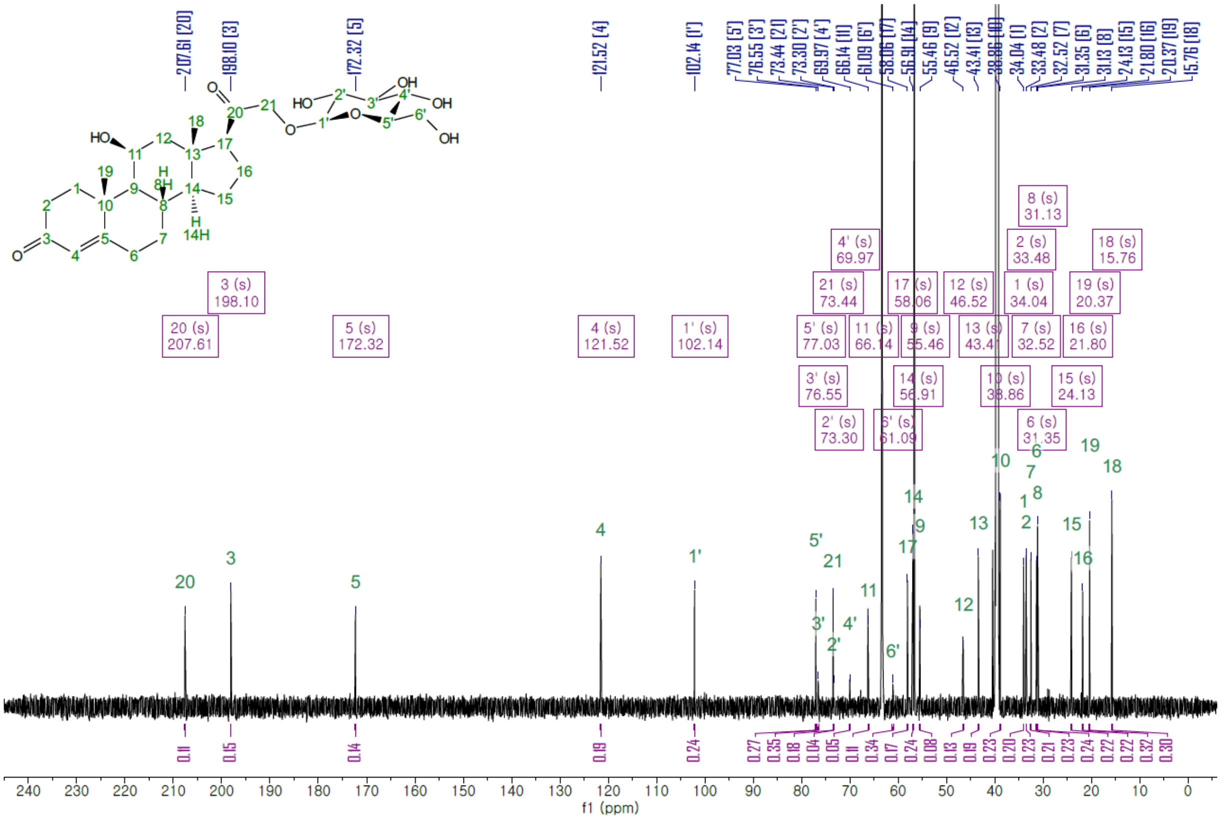

Figure S1.

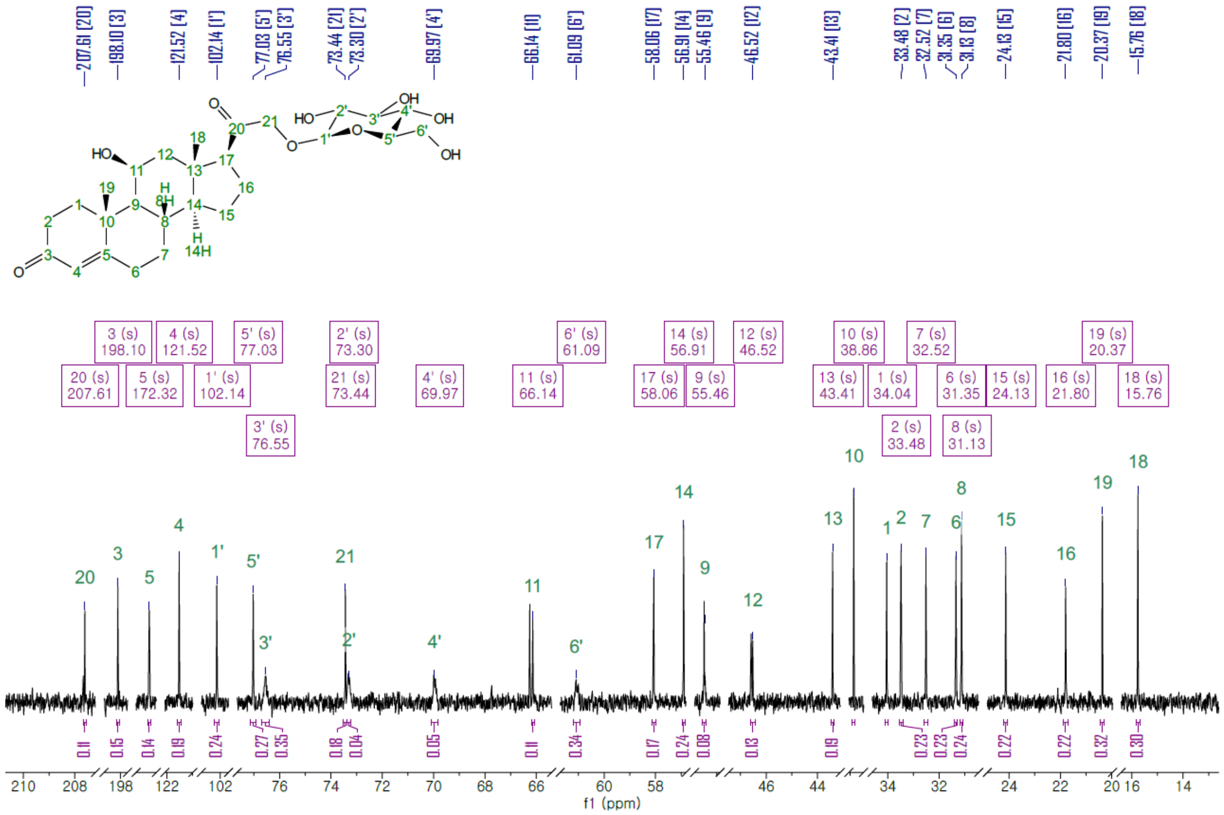

Figure S2.

^{1}H-^{1}H COSY

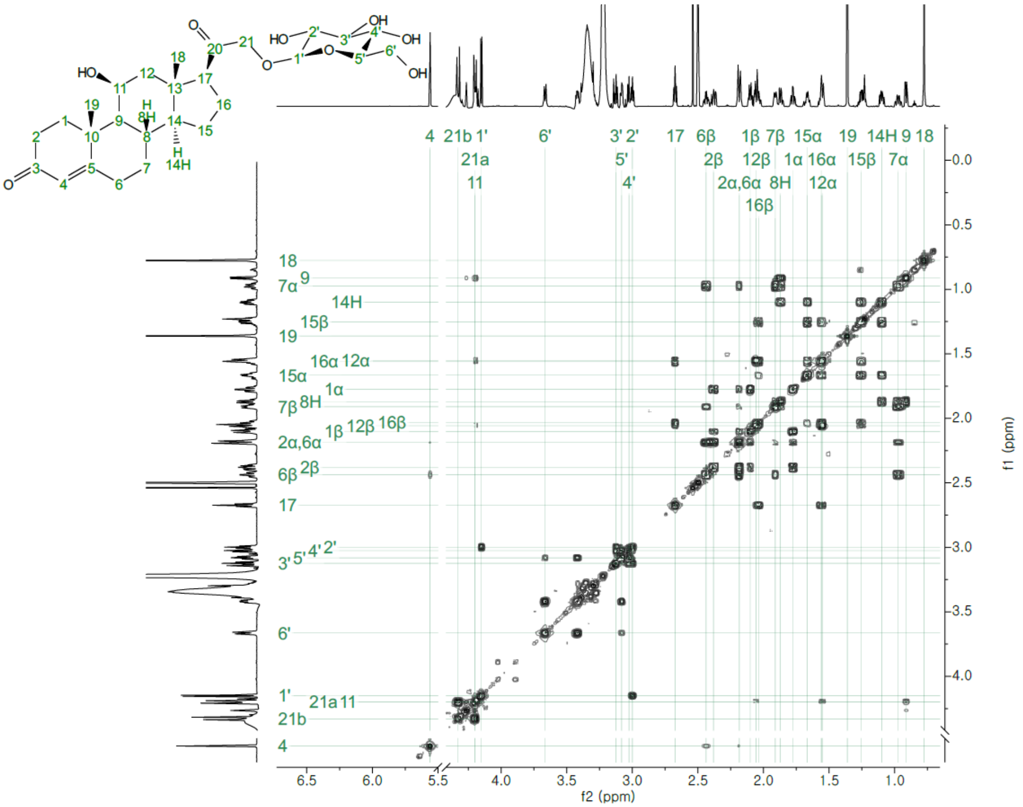

ROESY

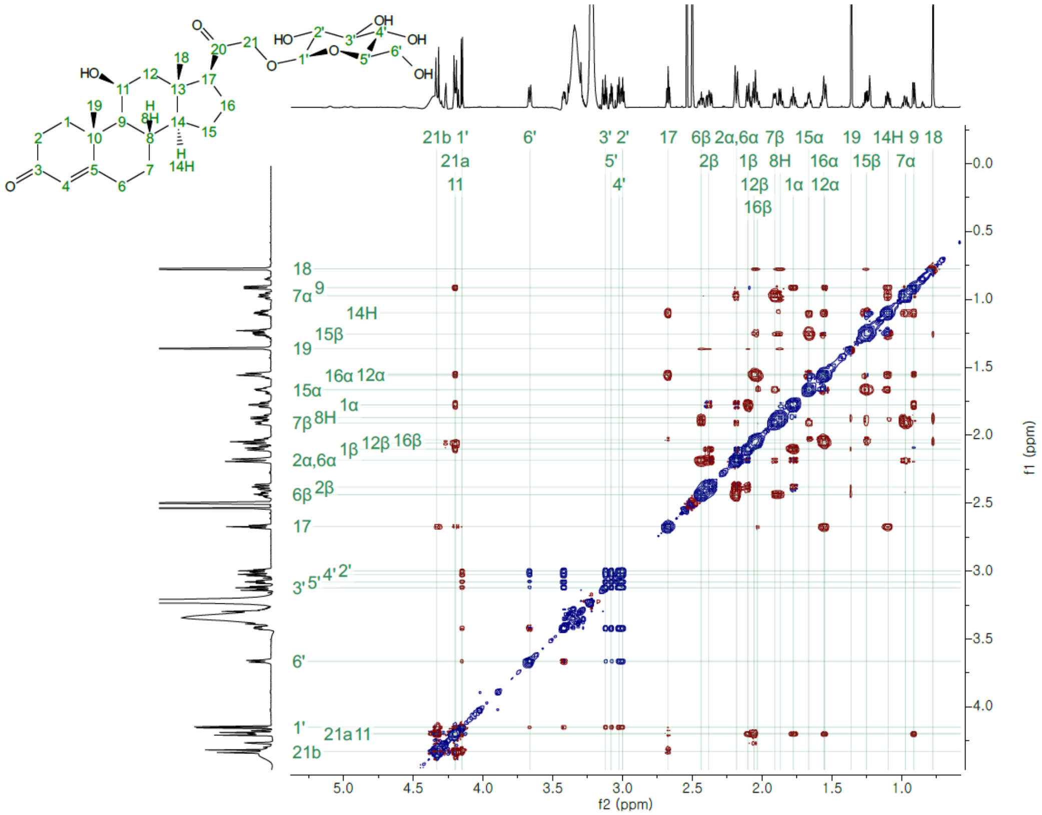

Figure S2.

HSQC-DEPT

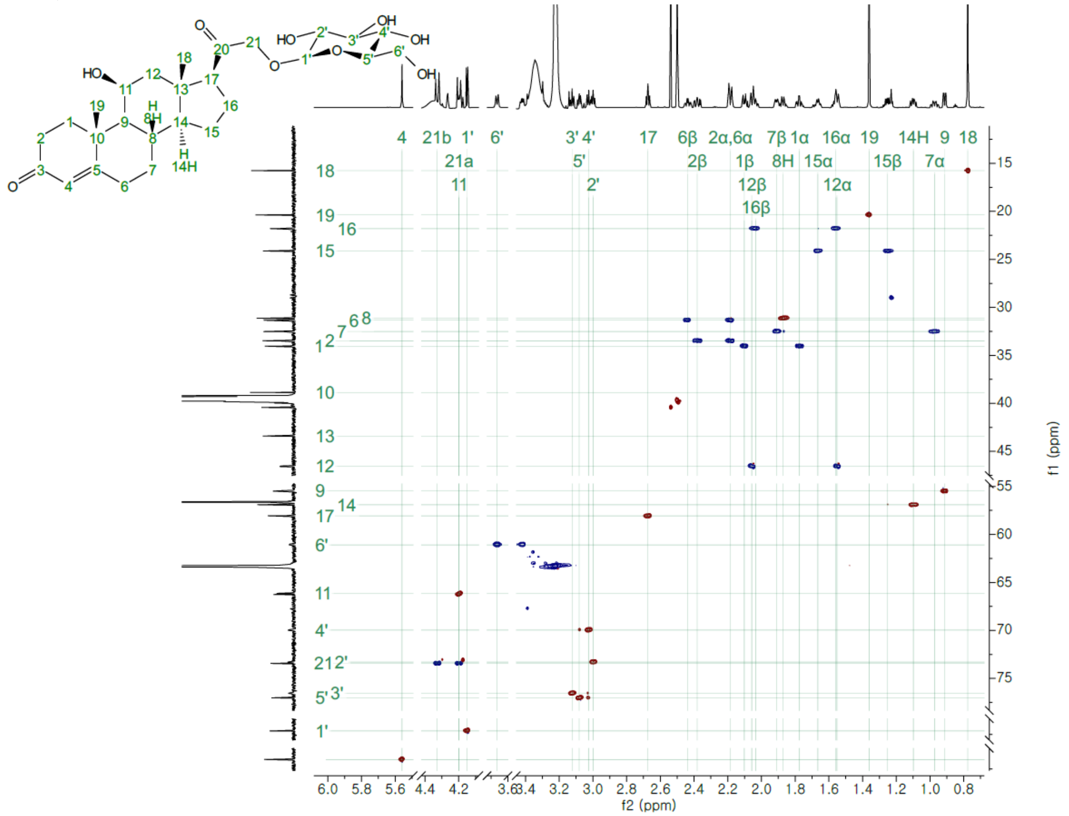

HMBC

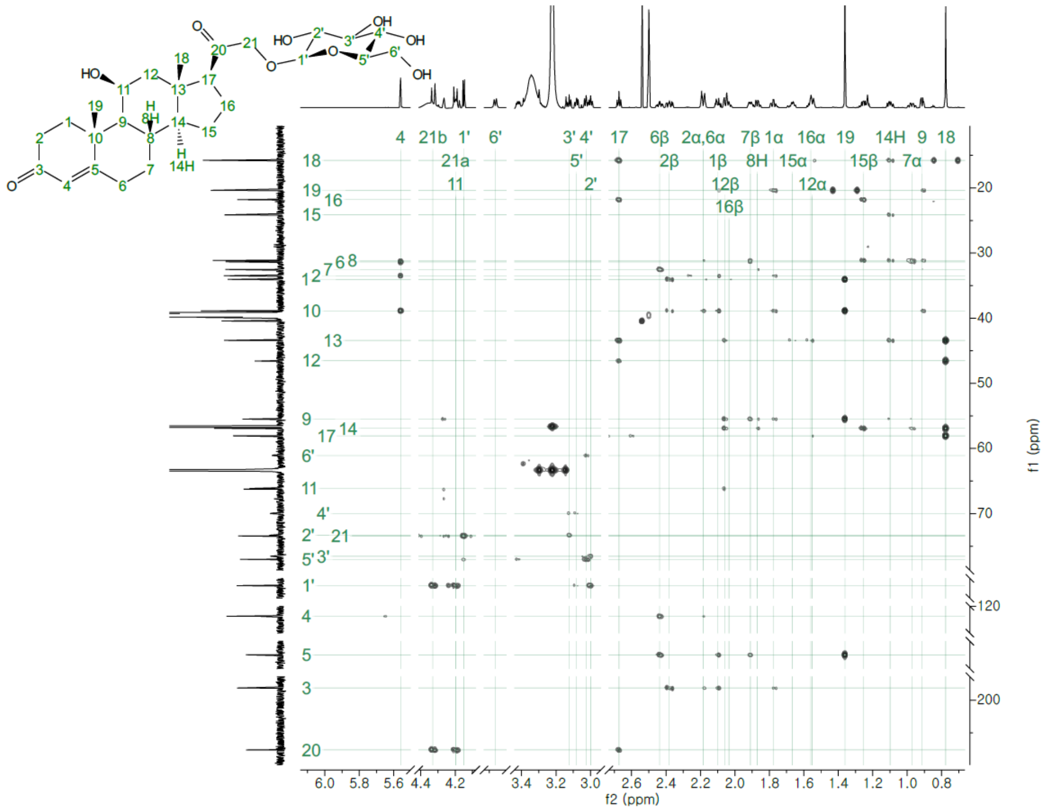

Supplement: Supplementary file 1 [file molecules-23-01783-s001.pdf]
